# Using Constrained-Disorder Principle-Based Systems to Improve the Performance of Digital Twins in Biological Systems

**DOI:** 10.3390/biomimetics8040359

**Published:** 2023-08-11

**Authors:** Tal Sigawi, Yaron Ilan

**Affiliations:** Department of Medicine, Hadassah Medical Center, Faculty of Medicine, Hebrew University, Jerusalem P.O. Box 12000, Israel; talsigawi@gmail.com

**Keywords:** digital twins, digital health, variability, noise, complex systems, system biology

## Abstract

Digital twins are computer programs that use real-world data to create simulations that predict the performance of processes, products, and systems. Digital twins may integrate artificial intelligence to improve their outputs. Models for dealing with uncertainties and noise are used to improve the accuracy of digital twins. Most currently used systems aim to reduce noise to improve their outputs. Nevertheless, biological systems are characterized by inherent variability, which is necessary for their proper function. The constrained-disorder principle defines living systems as having a disorder as part of their existence and proper operation while kept within dynamic boundaries. In the present paper, we review the role of noise in complex systems and its use in bioengineering. We describe the use of digital twins for medical applications and current methods for dealing with noise and uncertainties in modeling. The paper presents methods to improve the accuracy and effectiveness of digital twin systems by continuously implementing variability signatures while simultaneously reducing unwanted noise in their inputs and outputs. Accounting for the noisy internal and external environments of complex biological systems is necessary for the future design of improved, more accurate digital twins.

## 1. Introduction

A digital twin is a computer program that uses real-world data to create simulations that predict how a system, a product, or a process perform [1,2]. These programs integrate artificial intelligence (AI) and software analytics to improve output [3]. In most currently used digital twins platforms, noise in input datasets detract from the accuracy of the results [4]. Different methods are used to reduce noise and uncertainties to improve the accuracy of program outputs [5].

In the present paper, we review methods for dealing with noise and uncertainties in digital twin systems and present several of their applications in biological systems. Variability is inherent to biological systems and is part of their normal function [6,7,8,9,10,11,12,13,14,15,16]. We introduce the concept of noise-dependent second-generation AI systems based on the constrained-disorder principle (CDP) to improve the performance of digital twins in biology. The paper describes the differentiation between noise, which is necessary for the proper function of biological systems, and unwanted noise, which detracts from an algorithm’s accuracy in improving the performance of digital twins for diagnosis, treatment, and predictions.

## 2. The Constrained-Disorder Principle Defines Noise as Inherent to Biological Systems

Biological systems are complex, and part of their complexity results from the inherent noise and variability that characterize their function. The constrained-disorder principle (CDP) defines biological systems as comprising a disorder within constrained random boundaries [6]. It defines living organisms as machines with a regulated degree of variability. Per the CDP, a disorder is necessary for the systems’ existence and proper operation [6,7].

Variability is inherent to all levels of biological systems [7,8,9,10]. At the genome level, variability characterizes normal DNA function, and a similar stochastic function is required for the proper function of RNA and proteins [8,9,10]. Fluctuations in gene expression, cell-to-cell signaling, and the cell environment are tightly regulated [11]. At the cellular level, multiple examples exist of the need for inherent variability. Dynamic instability characterizes microtubule function and implies variability in their elongation and shortening [6,12,13,14,15]. At the whole-organ function level, heart rate variability (HRV), blood pressure variability, and gait variability are examples of functions that require noise for the systems’ proper function [17,18,19,20].

## 3. Bioengineering Needs to Account for Variability

System engineering and computerized architectures of biological systems must account for the variability that characterizes them [16,21]. Engineering single-cell and multi-cellular biological systems using a combination of synthetic and systems biology, nanobiotechnology, pharmaceutical science, and computational approaches are challenged by noise and the intra- and inter-cellular fluctuations that characterize systems [22]. Bioengineering must comprise noisy variables inherent to biological systems and requires that biological noise is recognized as a design element with fundamentals that can be actively controlled [23]. As part of a stochastic design, engineering noise can improve modeling accuracy [16,17].

## 4. Digital Twins Use Real-World Data to Create Simulations

Digital twins were presented and defined by Grieves as a model, including virtual products, physical products, and their connection [18]. They use real-world data to create simulations that predict how a system performs. Digital twins reflect the real-time operation state, future evolution trends, and essential functions of systems by integrating historical data, real-time data, and physical models [19]. A digital twin is a virtual clone of a tangible entity, a vehicle engine, a person, or an intangible system, and is studied independently of its real-world counterpart to make informed judgments [20,24].

The definition provided for a digital twin differs from the conventional definition as a key tool for digital transformation in the manufacturing industry. According to the conventional definition, a digital twin is a virtual representation of a physical good, process or product. A DT is a virtual representation of a physical asset, process, or system that enables real-time monitoring, analysis, and optimization [25].

Digital twins collect data from multiple dimensions such as personnel, equipment, materials, processes, and the environment, generating an actual operation state in objects [26]. They conduct virtual simulations driven by real-time data to generate an optimal linkage operation strategy and process regulation [27,28,29]. Digital twins accurately describe and optimize the physical entity using an optimization model [30]. They make up for the deficiency of traditional modeling and simulation methods by reflecting the physical object’s essential characteristics [24,31,32].

The digital twin platform is divided into three linkage stages [24,33,34]. In the initial planning stage, digital twins collect real-time operation data on factors such as personnel, equipment, materials, methods, and the environment, creating a virtual object layer. In the dynamic revision planning stage, the virtual object layer in the digital twins-enabled architecture reflects the target. It dynamically evaluates and optimizes the process based on relevant models while comparing the actual operation state of the system with the dynamic optimization state. At the dynamic coordination and control stage, the feeding back of the results of the dynamic revision planning to relevant units in real time realizes the online adjustments of the system [24,33,35].

The virtual twin can adapt to changes in its physical counterpart, just as the physical object responds to interventions in the virtual twin [36,37,38]. Digital twins follow the coevolution of digital objects and physical entities by continuously collecting relevant data and improving themselves [31,39]. The model adapts using monitoring, collection, and processing of the associated sensors’ data on the system, enabling digital twins to make predictions about their corresponding physical counterparts [24,40]. Digital twins allow for forecasting and interventions to prevent problems under ever-changing real-world conditions [27,28]. The deviation between the digital twin’s prediction and the actual state warns of a problem [29]. Digital twins are self-improving as they continuously monitor the divergence between predictions and observations and use these discrepancies to improve their accuracy [41,42,43].

A digital twin focuses on manufacturing operations by gathering data from physical sources and information technology [44]. The engineering of digital twin services is challenged by the complexity of interactions and the heterogeneous nature of these services. The concurrent use of models and data (e.g., model-based systems engineering (MBSE)) is considered for complex systems in service-oriented engineering projects. It was recently proposed that recalling information systems can improve workflow among enterprises and servitization [44].

## 5. Using Digital Twin Systems in Biology

The design of a digital twin model in biology is based on selecting a specific purpose and identifying the components of the targeted biological system and the interactions between them [45]. It implies capturing the mechanisms and features relevant to the selected purpose and the possible interventions, generating a conceptual map of the model that integrates all pre-defined components [46]. The model is validated using human or other preclinical data. These steps are followed by uncertainty quantification of the model’s behavior [29].

A model’s personalization requires using the appropriate patient-specific data to generate a subject-specific digital twin [47]. The model inputs consist of single-time or repeated clinical and laboratory biomarker measurements during diagnosis and therapeutic intervention. The model output consists of binary outputs, i.e., whether to intervene or not, or dynamic outputs, such as changes over time from a predetermined set of health parameters [24,29]. The final model requires extensive testing under numerous conditions and the adjustment of its features and parameters to improve accuracy [24].

Digital twins in biology are data-driven, based on mechanistic computational models that use data that inform the models at the individual scales integrated into a comprehensive multiscale model [29]. Digital twins enable the construction of a “core” model that represents commonly shared features in a biological system, which can be extended and customized with additional parameters and personalized using data from an individual subject [29,48]. Modules combining plug-and-play methods, such as the Python-based architecture, ease the collaboration between centers to improve the datasets used and support interactions between several resources [36,49].

## 6. Applications of Digital Twins in Healthcare

The use of digital twins in healthcare is enhanced by improved computer capacity and the development of wearable and smart devices, which provide abundant data that require correct interpretation [37,38]. Nevertheless, implementing digital twins in medicine presents challenges due to the complexity and variability of biological processes, which are translated into noisy dynamic data [50].

Digital twins in healthcare provide advantages such as the remote visibility of patients and their internal organ systems and processes, and their physical devices’ behavior [51]. Digital twin models assist in drug development, early diagnosis, treatment optimization, and precision medicine [52]. Digital twins provide personalized medicine by bridging the inter-individual variability in the inputs and the response to treatment and disease trajectories [53]. They use individual cell, genetic, longitudinal clinical, and wellness data to produce distinct personalized models and collect continuous data on parameters from subjects and the environment. A virtual replica can test a therapeutic regimen for its twin’s illness, identifying the best-fitting treatment [41,42].

Virtual twins can identify a pre-illness condition, enabling preventive measures to be taken [43,54]. The historical and real-time data of individuals and the population assist machine learning (ML) algorithms in predicting future outcomes [55,56,57]. An example is a virtual representation of a single person where every known medicine for that subject’s illness is tested, enabling the improvement of therapeutic regimens [58]. The systems monitor the virtual “person” and provide notifications about side effects, enabling preventive action [54,59]. Historical and real-time data assist ML systems in predicting future conditions [55,60,61]. Models are generated for predicting the efficacy of a particular treatment based on frequent measurements of a patient’s clinical or laboratory biomarkers, or “offline”, using simulated patient populations for developing new drugs [29]. Using digital twins enables the exploration of the effects of treatments in an individualized manner while searching for personalized biomarkers [48,52].

In cardiology, digital twins can improve planning and decision-making in cardiac interventions by creating individual structural and functional heart models [37,43,62]. The models simulate drug impact and responses to the implementation of devices, and can refine their output based on real-time intraoperative data [37,63]. This method applies to cardiac resynchronization therapy, valve replacement surgeries, catheter ablation procedures, and the correction of congenital heart diseases [37,63,64]. For patients with heart failure who require pacemakers designed for cardiac resynchronization therapy (CRT), a digital twin of the patient’s heart uses data from MRI, ECG, and blood pressure monitoring, assisting in defining the position of the pacemaker lead before surgery [65,66]. A digital twin was designed as a virtual three-dimensional model of the coronary blood to calculate the fractional flow reserve as an alternative to cardiac catheterization and assess the severity of carotid artery stenosis based on head vibrations [67]. Another regulatory-approved digital twin is that of an arterial aneurysm and its adjacent vasculature. It optimizes endovascular interventions by performing multiple simulations of endovascular implants during the procedure based on an angiography image [68].

An artificial pancreas for treating type 1 diabetes mellitus comprises a closed-loop system that incorporates real-time glucose levels into an algorithm that directs insulin delivery [69]. It contains several features of digital twins, including collecting and analyzing patient-specific online data and generating clinically meaningful outputs [70,71,72]. For patients with type 2 diabetes mellitus, data on blood sugar levels, vital signs, lifestyle, and daily nutritional habits are incorporated into a model that generates recommendations regarding dietary modifications and drug prescriptions [73,74,75]. The model follows weight reduction, improved glycemic control, and insulin sensitivity, reducing the need for anti-diabetic medications [73,74,75,76].

In oncology, digital twin systems are developed for predicting outcomes and optimizing therapies [62,77]. Digital twins of the immune system have been developed while facing the challenge if its inherent complexity and the difficulty of measuring multiple variables of a patient’s immune state [29]. These models represent numerous autoimmune, inflammatory, infectious, and malignant diseases [62]. Digital twins were introduced as a tool for patients with multiple sclerosis to improve diagnosis, monitor disease progression, and adjust therapy [78]. Systems have been developed for modeling inflammatory bowel disease [79]. Digital twins have emerged in infectious diseases, driven by the coronavirus pandemic. These systems integrate patient-specific clinical data with computer simulations of the viral infection and immune response to produce predictive outcomes and guide treatment [55,80].

In orthopedics, a digital twin of the human vertebra, simulating its structure and response to physical stress, predicts the risk of fractures in predisposed subjects [64]. A limb model simulating its anatomy and range of motion facilitates planning and improves the outcome of arthroplasty procedures [57,81]. A digital twin of long bone fractures simulates stabilization modalities to guide intervention and postoperative management [79]. These applications are extended to other surgical domains for planning and training for invasive procedures and predicting complications [55,60].

The use of digital twins for image and pattern analysis is being developed to interpret CT or MRI images and describe drug absorption distribution metabolism and elimination [42,82]. Digital twins can be used for designing virtual representations of medical facilities or services, for the pharmaceutical industry, and for educational purposes [37,61].

While promising, these models suffer from a lack of accounting for the inherent noise of biological systems and the difficulties of dealing with unwanted noise and uncertainties.

## 7. The Need to Model Uncertainties and Noise in Complex Systems

Despite the achievements of the digital twin systems, uncertainties are an integral part of the inference process [38,68]. Uncertainties can result from multiple structural, parametric, algorithmic, and observational variables. If these uncertainties are not adequately addressed, the allegedly optimal solutions or predictions generated by the model may fail in real life [41]. Inaccuracy or uncertainty in biology may cause misleading inferences and inadequate decision-making, potentially jeopardizing a patient’s health [37]. Confidence in prediction is also valuable for establishing clinicians’ trust in new technologies [38,68].

Digital twins can be designed to deal with the uncertainty and unpredictability that are part of the life cycle of complex systems [83]. Uncertainty quantification of digital twin models is necessary to improve their accuracy under dynamic internal and external environmental conditions. The current models aim to estimate and reduce the effect of uncertainties on model predictions [29,83,84].

Uncertainties in medical digital twin systems arise from the inherent complexity and variability of biological processes, which are reflected by the inaccuracy of the computational models [41]. The two primary sources of uncertainty that have been described are ‘aleatoric uncertainty’ and ‘epistemic uncertainty’ [41,85]. The former relates to statistical or data uncertainty and stems from unpredictable randomness, stochasticity, and the intrinsic noise of the measured variables [38,41,85]. This type of uncertainty is not reduced, even with more data collected [38,68]. Epistemic uncertainty refers to model or systematic uncertainty. It originates from the structure and parameters of the mathematical algorithms used for data analysis, including their assumptions and approximations, and from missing values and errors in the measurements [41,68,70,85]. It reflects incomplete or inadequate knowledge and can be reduced by adding data to the system [41,71,85].

These two types of uncertainty reflect the differences between noise and variability that characterizes biological systems for which models need to account, and the unwanted noise that results from a lack of data, inaccuracies in measurements, and confounding variables in the data.

As each subject changes over time concerning its inherent noise, as determined by the CDP, the model requires periodic recalibration to maintain its relevance. This is an ongoing learning process that augments the model’s performance [44]. Incorporating machinery for continuous model improvement, where deviations between model predictions and outputs and actual observations are followed, can refine model parameters and reduce uncertainty [29,72]. In addition, each time a digital twin model is used, all similar digital twin models are improved based on the learned experience [48,78].

## 8. Digital Twins’ Methods for Dealing with Uncertainties

Neural network (NN) decisions are unreliable because they lack expressiveness and transparency [73]. An NN cannot understand or resonate with the content of the data it is trained on and cannot explain its decisions [74,75]. NNs are sensitive to small data distribution changes, making it difficult to rely on their predictions, and they show overconfidence and are vulnerable to adversarial attacks [76,86]. Several methods have been applied to medical deep learning systems for identifying and quantifying uncertainties, including Bayesian inference, fuzzy systems, and ensemble methods [41].

Considering uncertainties during data processing provides better verification and validation of the output and improves the system’s reliability [38,41,85]. Several Bayesian inference methods are explored:

i. Complete Bayesian analysis is a component of probability statistics derived from the Bayesian theorem used for uncertainty quantification [41,87]. Bayesian inference estimates the probability of a hypothesis under updated knowledge (i.e., posterior probability). It uses prior probability (the probability of the hypothesis occurring irrespective of the updated knowledge), model evidence (the observation of experimental or simulated data), and likelihood (the probability of specific parameters being observed if the hypothesis is correct) [85,87]. Under the Bayesian principles, a prior distribution for the uncertain parameters is assumed based on expert knowledge. Using model evidence, the posterior distribution of these uncertain parameters is estimated via the formula, and a confidence interval reflecting the reliability of the result is extracted [38,68,85,87]. As more evidence accumulates in subsequent simulations, the parameters are updated, and the posterior distribution shows improved accuracy [41]. Combining the Bayesian approach with deep learning is helpful for uncertainty quantification, providing a framework for the training process, Bayesian deep learning [41,87].

These systems learn a distribution over each of the network’s weight parameters instead of using deterministic single-point weights, and optimize the network by averaging all possible weights [38,68]. This enables the estimation of all uncertainties associated with the predicted output and yields a higher value in cases of insufficient data [68]. These methods are used in medical digital twins to improve their prediction capabilities, guide the timing of interventions, and enable early diagnosis [38,52,68,71,88,89,90].

ii. The Markov Chain Monte Carlo (MCMC) method is used to estimate the posterior distribution, which is computationally intensive and sometimes cannot be calculated analytically [41,68]. MCMC addresses the sampling problem via probability distribution and approximation methods (e.g., Variational Inference and Monte Carlo dropouts) [68]. Monte Carlo (MC) simulations attempt to predict all the possible results of a system with random variables [41]. The algorithm runs multiple possible values within the known range of each input parameter, producing an output of a probability distribution that reflects every possible result and its likelihood [70]. The MCMC method enables the expression of the posterior probability of complex real-world processes by using computer simulations of random samplings from the probability distribution [87]. MCMC is generated within the space of all possible results. The progression from one possible value to the next is random, but using different algorithms, it is set up so that values derived from more plausible models appear more frequently [87]. This process approximates the most probable results and achieves more accurate results as more samples are obtained [70].

MCMC is the most frequently used sampling method for Bayesian inference and can be used when the analytical calculation of a posterior distribution is impossible or laborious [41,68]. However, its application in deep learning models tackles computational difficulties due to a need for multiple iterations to calculate the posterior probability, resulting in scarce use in deep learning in medicine [68]. For this reason, approximation algorithms for sampling distributions have been developed, enabling the application of deep learning techniques in large and complex databases, although they generate less accurate results [68].

iii. Variational inference (VI) for approximate Bayesian inference provides a computational approximation of the intractable posterior probability distribution by solving an optimization problem and finding a tractable distribution similar to the unknown one [68,70]. VI is faster than MCMC, and the convergence into a result is unequivocal [68]. However, it involves complex calculations, approximates the desired distribution rather than the theoretically optimal solution with considerably fewer samplings, and is applicable to large-scale datasets and complex models [68,70].
iv. The Monte Carlo dropout method for approximate Bayesian inference prevents overfitting during the training of deep learning systems, improving generalization and prediction abilities from unseen data during the testing phase [68]. Some neurons within the hidden layers of a deep NN are randomly omitted, including their incoming and outgoing connections, resulting in diminished network complexity. As the neuron elimination is random, each training iteration is performed on a different edited network, resulting in multiple predictions generated from the same data. The output is a distribution of predictions produced by ensembles of smaller networks, reflecting the model’s uncertainty [38,70]. This improves the system’s performance by capturing randomness and quantifying uncertainties [38].

A fuzzy inference system represents inaccurate data for uncertain or approximate reasoning and is not derived from the probability theory [91,92]—a fuzzy method models a system with many unknown parameters and deals with epistemic uncertainties. Fuzzy logic encodes vague values from 0 to 1, representing a degree of truth, in contrast to traditional binary computer logic, enabling the encoding of a more complex representation of reality, resulting in a more accurate output [41,91]. Distinct values are converted into fuzzy variables, representing a degree of membership of a specific value to linguistic categories according to membership functions, ranging from 0, meaning not belonging to the fuzzy set, to 1, ultimately meaning belonging. Fuzzy logic rules are applied to these variables (i.e., inference) to create new fuzzy variables, which are converted back into crisp values (i.e., defuzzification) using functions of the desired output [41]. Integrating fuzzy logic concepts into artificial NN architecture results in a hybrid system termed a ‘neuro-fuzzy system’ [92]. Fuzzy systems are applied to the early detection of chronic diseases, in the treatment diabetic patients, and in artificial pancreases [41,82,92].

Ensemble methods combine predictions from several independent models of deep neural networks, ensemble members, to generate an output [70]. This integration reduces the model’s uncertainty, improves its accuracy, and quantifies its uncertainty by examining the variance between the members’ predictions [70]. The limitation of ensemble methods is the increased computing power and time required for simultaneously testing different models [93,94,95,96,97,98,99,100,101,102,103,104,105,106,107,108,109,110,111,112,113,114,115,116].

An example of dealing with uncertainties includes an approach for personalizing biophysically active models using a two-step multi-fidelity solution to reduce uncertainty in digital twins in cardiology [83]. In the first step, dynamic mechanical behavior in a given 3D electromechanics model is represented by a personalized low-fidelity model via calibration to clinical cavity pressure data. In the second step, median traces of nodal cellular active stress, intracellular calcium concentration, and fiber stretch personalize the model at the cellular scale, creating a cardiac electromechanics model. The algorithm’s robustness against uncertainty in the clinical data and variations in the initial guesses are shown in the validation study [83].

Optimizing digital twins under uncertainty in nuclear power systems is based on maximizing the information gain and performance of the physical asset [84]. Model-free techniques are adopted to augment limitations in the model-based approaches. The incorporation of uncertainty quantification (UQ) enables the propagation of uncertainty from digital representations to predict the behavior of the physical asset. Inverse UQ allows for the incorporation of data from new measurements, obtained from the physical asset, into the digital twin [84].

Traditional optimization algorithms are based on a single initial value, making the process cumbersome. A genetic algorithm (GA) is inspired by the process of natural selection in evolutionary algorithms (EA), and is used for designing digital twins [117]. A GA relies on biologically inspired operators such as mutation, crossover, and selection. Biological individuals with strong adaptability have a high probability of survival against a dynamic environment using better genes [118,119]. Using a GA overcomes problems by determining the optimal solution to problems through repetitive genetic operations [120]. Increasing fitness improves the individual gene, bringing it closer to the optimal solution [121]. GA fitness implies adaptability to the environment and solves problems by referring to chromosomes made of genes, implying the evolutionary advantage of selected genes [122]. A GA requires the conversion of problem parameters into chromosomes in a coding process and the conversion of GA individuals into solutions in a decoding process [123]. A GA eases the solving of problems using population search and probability for searching for a specific population. It randomly searches for the problem solution, improving optimization efficiency [124,125]. A GA uses a fitness-based search by constructing the fitness function and determining a search range and direction according to fitness [126].

The above-described methods provide tools for dealing with noise and uncertainties; nevertheless, they oversimplify the challenge of complex biological systems by ignoring the inherent noise required for proper function and the need to personalize the noise. Using means and distributions in analyzing these systems may be associated with biases that ignore the dynamicity of these systems and the need to personalize the outputs [9,93,127].

## 9. Improving Digital Twins for Biological Systems by Differentiating between Inherent Noise and Measurement-Related Unwanted Noise

The computerized architectures of biological systems must account for systems’ inherent noise [6]. This requires differentiation between these systems’ inherent noise and noise resulting from the uncleanliness of datasets and noisy measurements. This differentiation is necessary for improving output accuracy. As the output characteristics of every system need to comprise its noise, this implies that the exact type of noise needs to be part of the output.

The CDP implies that every system is characterized by a constrained-disorder bounded by dynamic boundaries [6,7,128]. Thus, differentiation between the two types of noise and uncertainty is necessary for generating accurate outputs using digital twins and is a critical element of their performance in complex biological systems in a personalized way [129].

The methods described above use approximations and distributions, which are beneficial for learning about systems and determining their trajectories. However, these methods are insufficient to reach the maximal accuracy required for analyzing dynamically disordered internal and external environments in complex biological systems [9,93,127]. Approximations and distributions are sufficient for an overall analysis of systems but may be insufficient for establishing personalized patient-based diagnoses, treatment plans, and outcome predictions. Not accounting for noise can lead to biases in the outputs of digital twins designed for generating treatment regimens. As noise is dynamic in a personalized way, ignoring it can lead to bias.

## 10. Augmented Digital Twins Make Use of Noise to Improve the Performance of Biological Systems

Second-generation AI systems are developed to use the inherent noise of biological systems to improve model accuracies and, therefore, diagnoses, response to therapies, and outcome predictions [113,130,131,132]. Based on the *n* = 1 concept, where the model generates subject-tailored outputs, these systems are dynamic, comprising methods that account for continuous alterations in the inherent noise of biological processes in a personalized way [93,133,134].

An example is the use of these systems to overcome the loss of response to chronic medical interventions. Partial or complete loss of response to chronic medications is a significant obstacle to achieving the long-term benefits of treatment in patients suffering from chronic diseases [133,135,136]. Regular dosing regimens are often associated with developing drug tolerance and loss of responsiveness. Digital twins designed for selecting the ideal therapy based on a large patient dataset are inadequate for resolving this problem as they do not account for the personalized dynamic noise that characterizes the dynamic response of a subject to a drug, which is dependent on multiple changing hosts and environmental variables [136].

Second-generation AI systems, which quantify signatures of biological variabilities and implement them into treatment algorithms dynamically, were proposed for overcoming the loss of response to medications [16,137,138,139,140,141,142,143,144,145,146,147,148,149,150,151,152,153,154,155,156,156,157,158]. Second-generation algorithms were found to account for dynamicity in response to therapies that characterized each subject [135]. This is based on evaluating the clinical outcome as an endpoint for the algorithm, which is the most relevant parameter for patients and healthcare providers. Digital twins that comprise the relevant noise-based signatures, such as HRV, or variability in cytokines secreted by immune cells in inflammatory disorders, provide higher accuracy for establishing diagnoses, generating treatment plans, and predicting outcomes dynamically in a personalized way [113,130,131,132].

In patients with chronic heart failure and diuretic resistance, a CDP-based second-generation AI system improved clinical and laboratory outcomes and reduced hospitalizations. Similar results were demonstrated in patients with chronic pain and multiple sclerosis [159].

Second-generation AI systems are an example of augmented digital twins that can improve biological systems’ performance by incorporating noise in a subject-tailored way.

**Figure 1:**
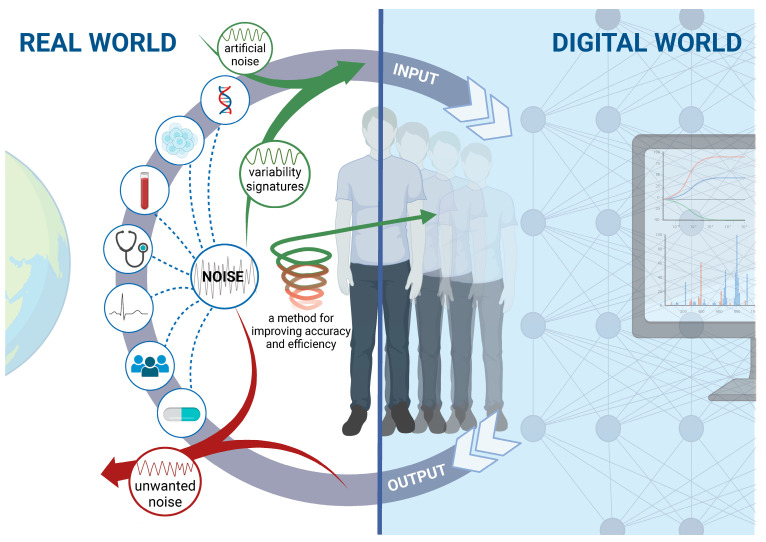
A schematic representation of methods for increasing the accuracy and effectiveness of digital twins in biological systems. Augmented digital twins’ architectures require personalized variability signatures while continuously adapting the models to changes in internal and external noisy environments. In parallel, digital twins are required to reduce the amount of unwanted noise and uncertainties in their inputs and outputs, including noise that results from the measurements themselves.

Figure 1 shows how the proposed digital twin system quantifies biological variabilities and inserts them into the digital twin algorithm in a personalized and parallel way, reducing unwanted noise and uncertainty.

## 11. Challenges Faced by Augmented Digital Twins in Medicine

The augmented digital twin architecture that accounts for the noise that characterizes biological and other complex systems while reducing unwanted noise, including noise that results from noisy measurements, impure datasets, and confounding variables in the input data, raises several questions.

In biological systems, the number of variables contributing to noise is endless and cannot be recognized in most cases. Accounting for those noisy variables that can be measured improves the algorithm output but can never reach complete accuracy. This means that an entirely accurate outcome may be unreachable. Nevertheless, implementing any degree of noise into the algorithm, such as treatment regimens, can improve its performance [16,137,138,139,140,141,142,143,144,145,146,147,148,149,150,151,152,153,154].

Attempts to improve accuracy using better measurement tools and multiple repetitions for inputs can improve the output, albeit insufficiently to obtain an entirely accurate output in biological systems under continuously changing conditions.

The amount of noise considered sufficient, based on personalized noise quantification, and in parallel, the amount of unwanted noise that is “small enough” not to detract from the model accuracy, can be validated using clinically meaningful outcome measures. Many biological systems lack tools to account for all the random parameters to be inserted into a model. This raises the question of whether implementing a fully randomized treatment regimen that is not personalized and is not based on the quantification of signatures of variability, can achieve a satisfactory result.

Per the CDP, the degree of randomness continuously changes [6]. Creating dynamic augmented digital twins that continuously modify their outputs in a personalized way requires the algorithm to have a high-speed response rate. This may not be applicable when receiving patient measurements; even if wearables are used for continuous measurements, they may be insufficient to keep up with the rapid changes in the host, their disease, and their environment. A prominent British statistician, George Box, said, “All models are wrong, but some are useful” [160]. Augmented digital twins comprise un-personalized noise and can provide a degree of accuracy that is sufficient for some clinical settings [93,133,134].

In summary, noise is inherent to complex biological systems, making accounting for it in digital twins’ architectures necessary. The task of dynamically quantifying signatures of variabilities from biological processes while reducing unnecessary noise that detracts from systems’ functions represents a significant challenge in developing digital twins for biological systems and other complex systems. Improved accuracy requires the implementation of biological noise into models in a continuous personalized manner. This involves adopting the models to account for continuously changing internal and external perturbations. Future studies will shed light on models that could achieve more accurate, augmented digital twins.

## Abbreviations

AI: artificial intelligence; CDP: constrained-disorder principle; ML: machine learning; NN: neural network; HRV: heart rate variability.
